# A Minimally Invasive Approach to Managing Isolated Gingival Recession

**DOI:** 10.7759/cureus.37195

**Published:** 2023-04-06

**Authors:** Vinitha Nair, Ram Sabarish, Deepak Ravindran, Balaji SK

**Affiliations:** 1 Department of Periodontology, Sri Ramachandra Dental College and Hospital, Sri Ramachandra Institute of Higher Education and Research, Chennai, IND; 2 Department of Periodontology, Sri Ramachandra Dental College and Hospital, Sri Ramachandra Medical College and Research Institute, Chennai, IND

**Keywords:** gingival recession/classification, gingival recession/surgery, connective tissue/transplantation, young adult, treatment outcome, surgical flaps

## Abstract

Gingival recession (GR), the apical shift of the gingival margin, results in root surface exposure. Its etiology is multifactorial, including teeth position within the dental arch, bony dehiscence, alveolar mucosa thickness, incorrect toothbrushing, orthodontic treatment, and periodontal disease. Coronally advanced flap (CAF) with a subepithelial connective tissue graft (SCTG) is the gold standard for managing GR. With the introduction of minimally invasive surgery, various techniques proposed for managing GR minimize patient morbidity and maximize surgical outcomes. The present case report is that of a 26-year-old male patient with the primary complaint of sensitivity in the upper right and left back teeth regions. Emdogain was used with SCTG for managing recession on the left side and with the xenogeneic collagen matrix (Mucograft) to cover recession on the right side. Post-operative healing was uneventful, with significant recession reduction and an increase in the width of the attached gingiva at both sites. GR, apart from posing as an esthetic complication, also results in tooth sensitivity. This makes the management of GR very important for which multiple treatment modalities are available. The current case report highlights the success of the minimally invasive tunneling technique in managing isolated GR.

## Introduction

Gingival recession (GR) is of aesthetic concern, and awareness about its management has increased in day-to-day clinical practice. Dental plaque and faulty tooth brushing are the most common factors associated with GR [1]. Its major consequence is root hypersensitivity, which can cause patient discomfort. Among the proposed range, Miller’s (1985) and Cairo’s (2011) classifications for GR are the most accepted. The World Workshop on Periodontics (2017) has adopted Cairo’s classification [1]. Several treatment modalities have been introduced to manage isolated GR. Complete coverage of recession defects associated with minimal probing depths and enhanced aesthetics is the goal of root coverage procedures [2]. Miller’s Class I and II (or) Cairo’s RT-1 and RT-2 recession defects have good prognoses following treatment [3]. Miller has classified GR into three classes. Class I represents marginal tissue recession not extending up to the mucogingival junction (MGJ), and class II is where the recession extends to or beyond the MGJ. Class III and IV represent cases where there is interdental bone and soft tissue loss with tooth malpositioning [3,4]. On the other hand, Cairo has classified GR into three types: recession type (RT) 1, 2, and 3. based on the assessment of clinical attachment loss (CAL) at buccal and interproximal sites. RT-1 represents no loss of interproximal attachment. RT-2 denotes the case where the interproximal attachment loss is less than or equal to the buccal attachment loss and RT-3 constitutes interproximal attachment loss greater than the buccal attachment loss [1].

Conventional techniques for the treatment of GR include using full or partial epithelialized free gingival grafts (FGG) and subepithelial connective tissue grafts (SCTG) in combination with various flap designs. With increased knowledge of minimally invasive surgery, tunneling techniques and their modifications have gained importance in GR management. One of the modifications includes the laterally closed tunneling technique by Sculean which aimed at achieving better root coverage and optimal soft tissue aesthetics [5]. The tunneling technique is advantageous because it involves an incision-free design, preserves the integrity of the interdental papilla, minimizes the risk of losing the papilla, maximizes the vascular supply to the underlying graft, and stabilizes the graft for optimal wound healing [2].

However, patient discomfort and donor site morbidity with conventional grafts have prompted researchers to develop new biomaterials for recession coverage, which match the efficacy of SCTG, optimizing treatment outcomes. One of these biomaterials includes Mucograft (GeistlichPharma, Wolhusen, Switzerland), a three-dimensional porcine collagen matrix (PCM) that has been in use for soft tissue regeneration [6,7]. Enamel Matrix Derivative (EMD) is another biomaterial used as an adjunct to soft-tissue grafting. EMD has been found useful in periodontal regeneration and proven to be effective and safe in improving clinical attachment levels, bone fill, and soft tissue healing. Studies have also reported that true periodontal regeneration could be achieved with the topical application of EMD [8].

The outcomes of recession coverage can be assessed using the root coverage esthetic score (RES) described by Cairo et al. in 2009 [9]. This system assessed five variables six months following surgery namely, the gingival margin (GM), soft tissue texture (STT), MGJ alignment, and gingival color (GC). Zero, three, or six points were given for the evaluation of the GM while a score of zero or one was given for the other variables. This case report highlights the efficacy of minimally invasive tunneling procedures in combination with Emdogain, SCTG, and Mucograft for the management of isolated GR in the maxillary right and left posterior region.

## Case presentation

**Figure 1:**
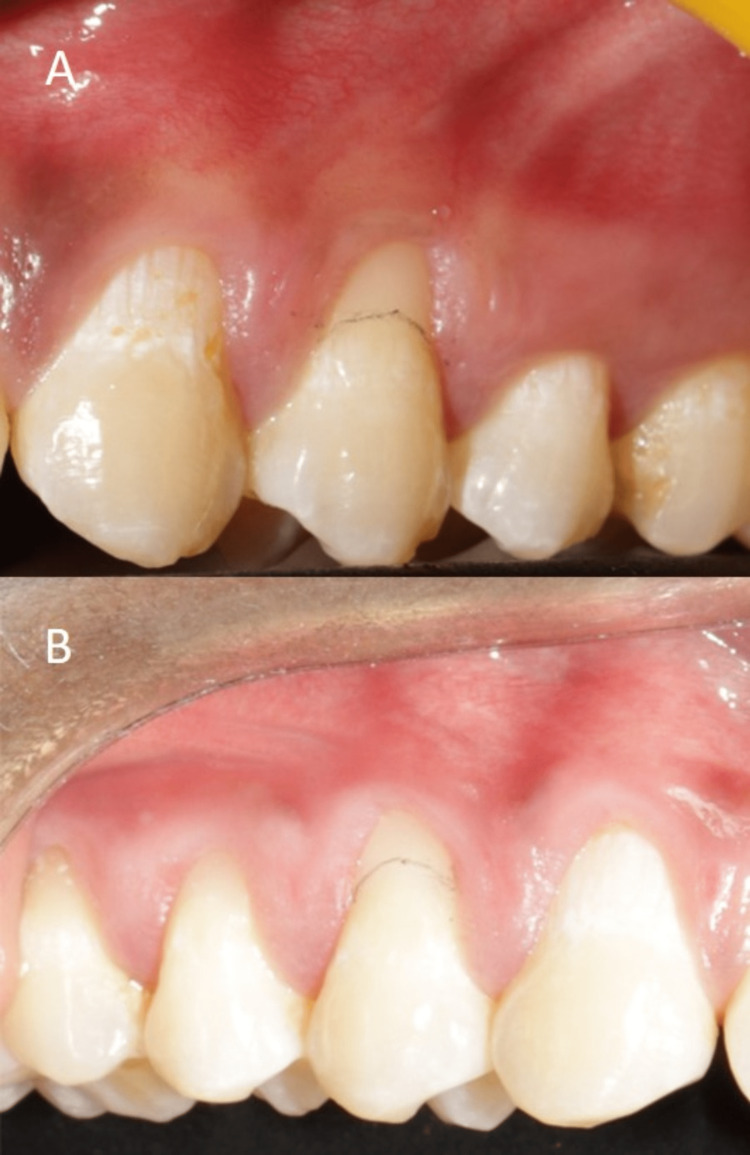
Pre-operative images of (A) 24, (B) 14

A 26-year-old male patient reported to the department with a complaint of sensitivity in the upper right and left back teeth region for two months. The patient had no relevant medical history. Intraoral examination revealed good oral hygiene. Periodontal examination revealed Miller’s Class I GR on the maxillary right and left first premolar. Applying Cairo’s classification of GR, both teeth had Cairo’s recession type 1 (RT-1) defect (Figures 1A, 1B). The possible etiology for GR in this case on both the right and left upper back tooth region would be faulty tooth brushing. The patient was counseled for the same, was given oral hygiene instructions, and advised proper tooth brushing technique using a soft-bristled toothbrush.

Both the teeth presented with 3 mm length and 4 mm width of recession. The width of the recession was measured at the level of the CEJ and the length was measured using CEJ as the reference point. There was no loss of interdental papilla (IDP). The gingival biotype was thin in relation to both teeth. The width of keratinized gingiva (WKG) at both sites was minimal (1mm) which was inadequate to maintain optimum function. However, as the patient complained of hypersensitivity, surgical root coverage was planned. Blood tests (Bleeding Time, Clotting Time, International Normalized Ratio) were performed and found to be within the normal range. The patient’s overall health was good with no local or systemic contraindications to surgical periodontal therapy. Written consent was obtained from the patient after an explanation of the entire treatment procedure.

Case management

Site 1

**Figure 2:**
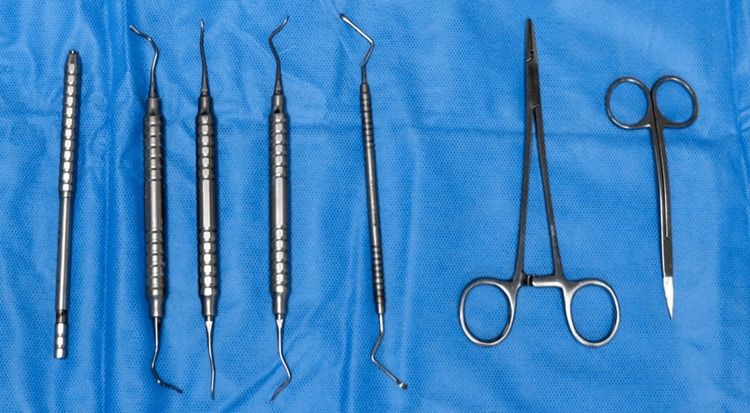
Tunneling instruments (Stoma)

The recession defect on the maxillary left first premolar, 24 was managed using the laterally closed tunneling technique described by Sculean et al. in 2018 in conjunction with Emdogain and SCTG [5]. The patient was prepared and adequate anaesthesia (2% lignocaine with 1:200,000 adrenaline) was administered with infiltration. Initial root preparation was performed by thorough scaling and root planning (SRP). The recipient bed was prepared by placing crevicular incisions using Swann Morton microsurgical blade No.67 (SM 67) on the affected tooth, including one tooth, mesially and distally. A mucoperiosteal tunnel was subsequently prepared using specially designed Sculean Aroca tunneling instruments (Stoma) (Figure 2).

**Figure 3:**
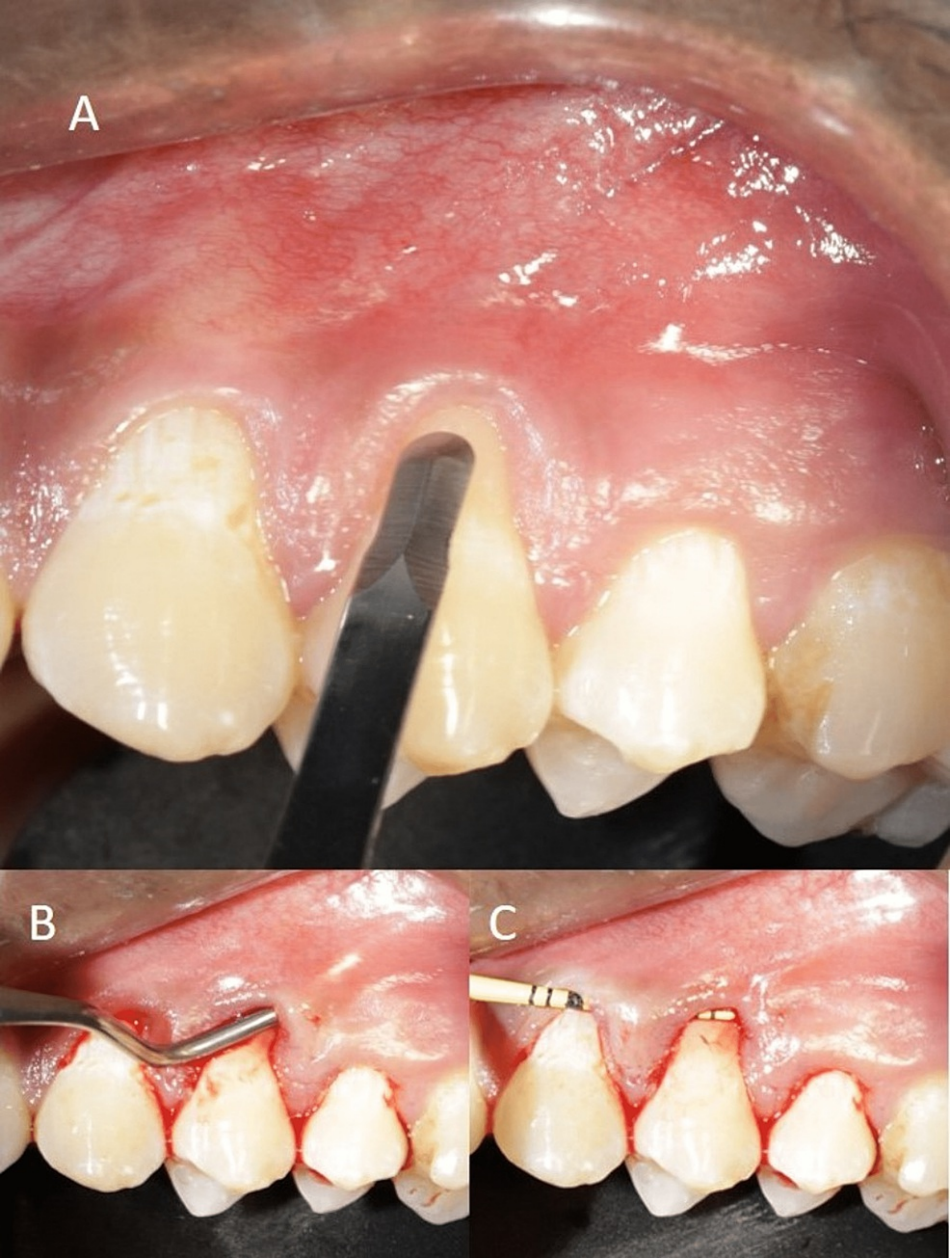
Tunnel/pouch preparation: (A) intrasulcular incisions placed using microsurgical blade and (B, C) tunnel preparation

**Figure 4:**
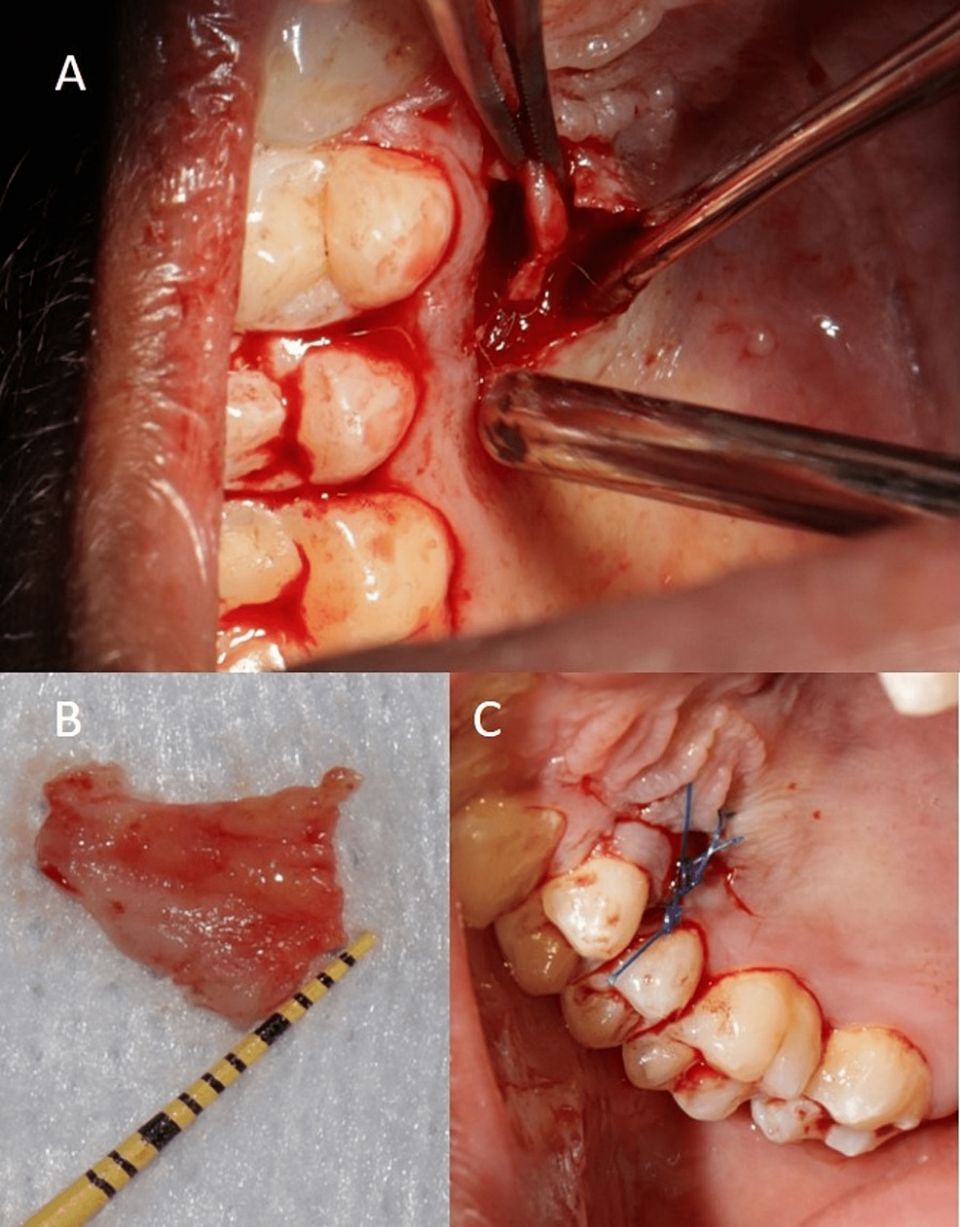
Harvesting of SCTG from palate using single incision technique (A) Harvesting of graft, (B) Procured graft, (C) Sutured donor site

Muscles and fibers, inserted laterally and apically, were released using microsurgical blades until a tension-free displacement of the flap margins was achieved (Figures 3A-3C). An SCTG of 1-1.5 mm thickness was harvested from the palate using the single incision technique [10]. The donor site was immediately sutured using 5-0 polypropylene sutures (Figures 4A-4C).

**Figure 5:**
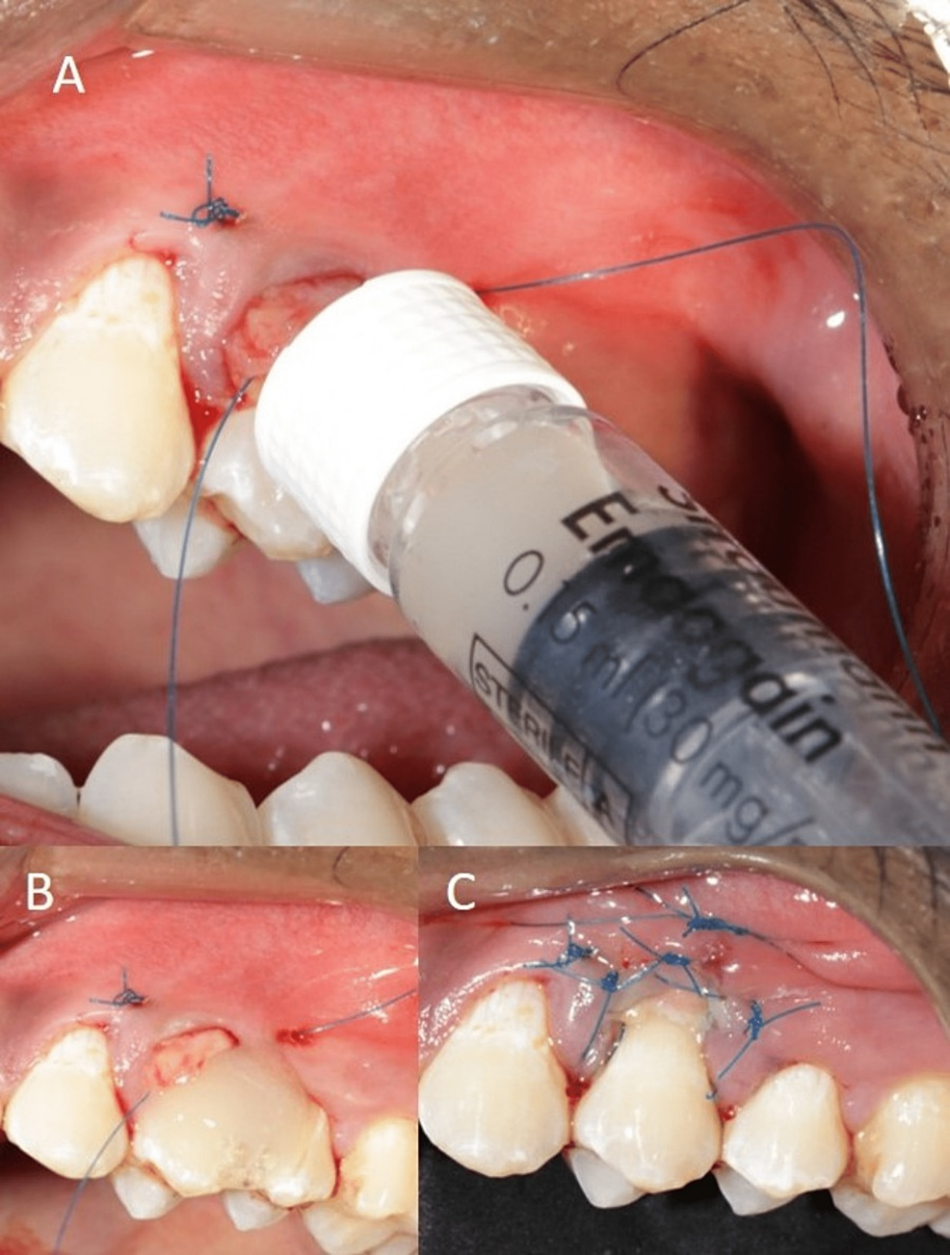
Recipient site preparation: (A, B) Emdogain application and (C) immediate post-operative image

**Figure 6:**
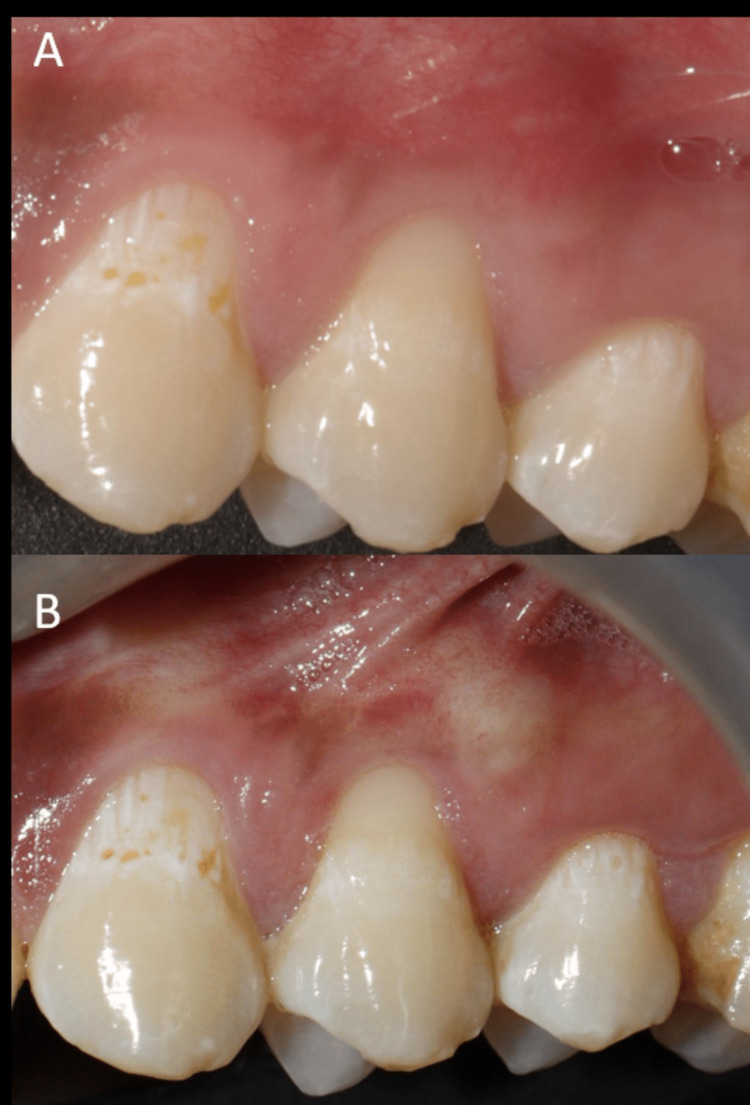
Post-operative images of 24. (A) Six-month post-operative image and (B) one-year post-operative image

The root surface was then conditioned with 24% ethylenediaminetetraacetic acid (EDTA) for 2 minutes (PrefGel, Straumann). Following rinsing with saline to remove the EDTA residue, Emdogain was applied to the root surface using an applicator tip (Figures 5A-5C). The SCTG was then passed through the tunnel and stabilized on the inner aspect of the pouch using mattress sutures. The graft was adapted to the level of the CEJ by sling suture using 5-0 polypropylene sutures. The margins of the pouch were then advanced to cover the graft and sutured to obtain tension-free coverage of the graft and denuded root surface. The patient was followed up for one year after the surgery. Partial root coverage was obtained with increased width of keratinized tissue at the site. Figures 6A, 6B show the six-month and one- year post- operative images of the site. The root coverage aesthetic score (RES) [9] for 24 was calculated, and a score of seven out of 10 was obtained.

Site 2

**Figure 7:**
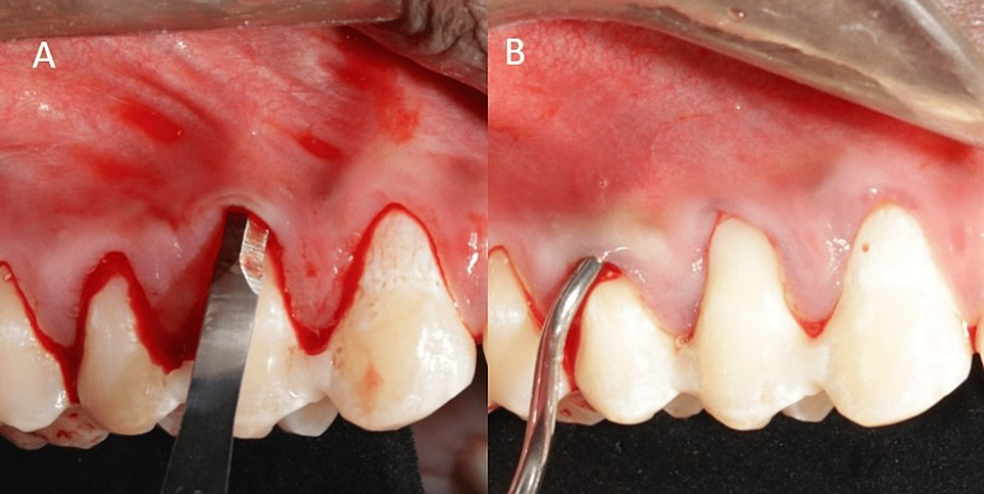
Pouch preparation i.r.t 14. (A) Intrasulcular incisions placed, (B) tunneling done using specialized instruments

The pouch and tunnel technique was performed for recession coverage on the maxillary right first premolar, 14 using Emdogain and Mucograft. Before surgery, composite stops were placed at the contact points to avoid collapse of the sutures at the interproximal spaces. After administration of adequate local anesthesia (2% lignocaine with 1:200,000 adrenaline), initial root preparation was performed by SRP. Sulcular incisions were placed on the concerned tooth as well as one tooth mesial and distal to it using a microsurgical blade (SM 67), tissues including the base of the IDP were gradually undermined which was extended up to the MCJ, so as to result in a freely mobile and tension-free pouch allowing easy placement of graft (Figures 7A, 7B).

**Figure 8:**
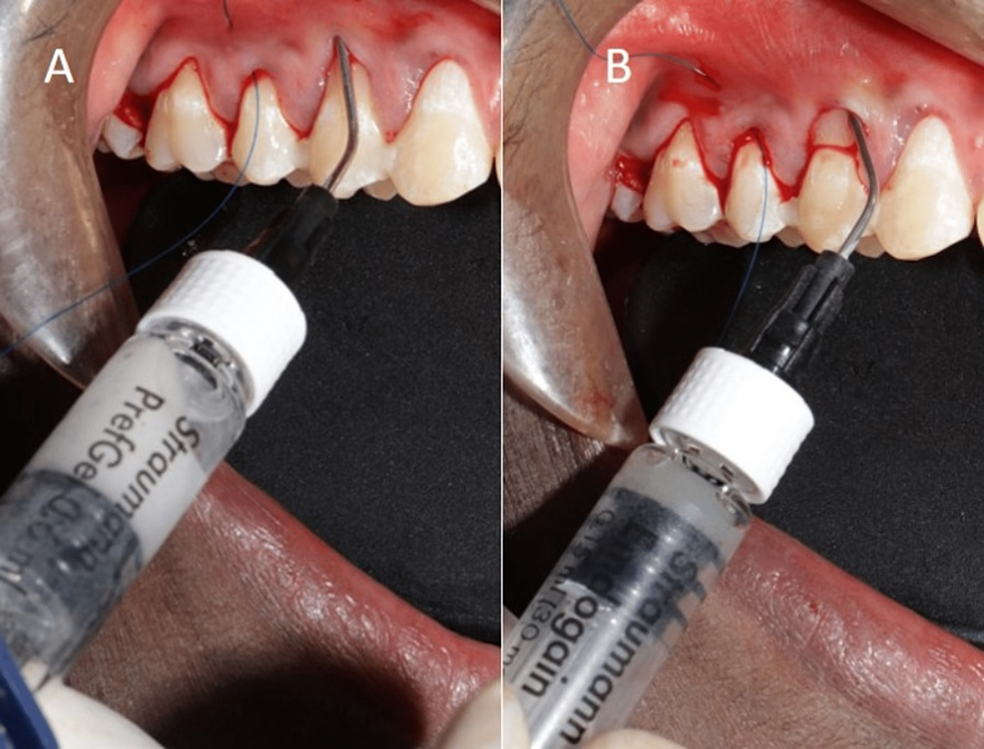
Recipient site preparation: (A) Pref Gel application, (B) Emdogain application

**Figure 9:**
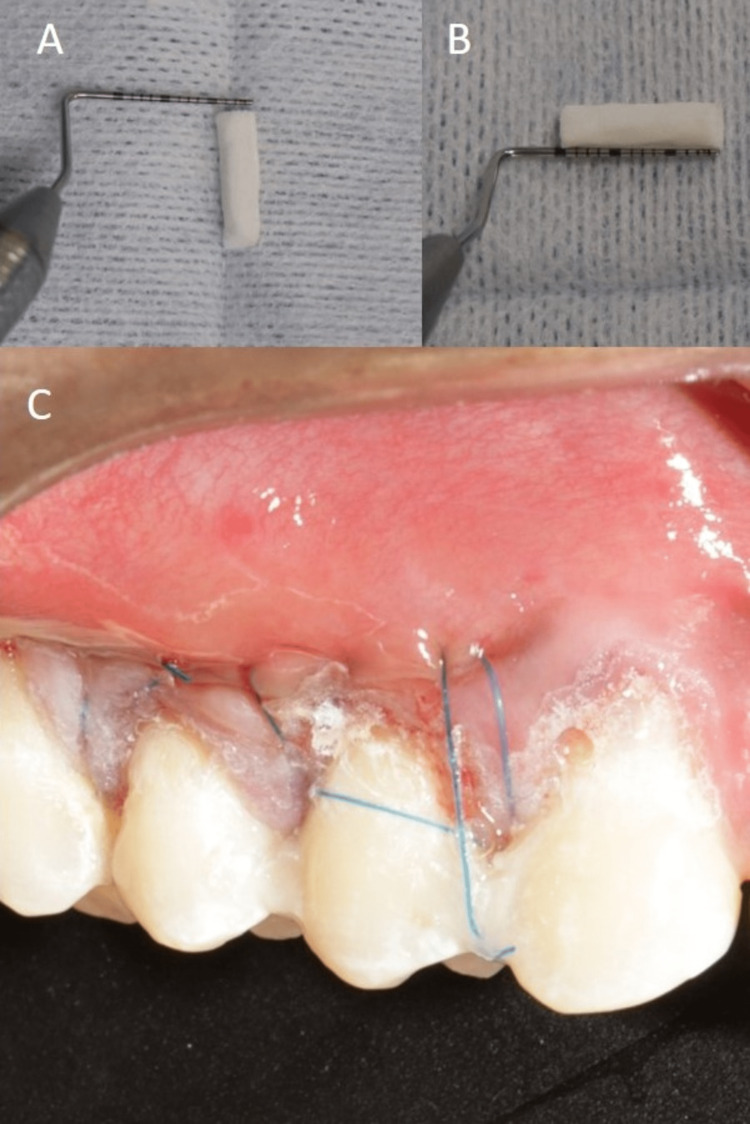
Mucograft placement into the recipient site: (A, B) Dimensions of Mucograft and (C) immediate post-operative image of the site with Periacryl (tissue adhesive) after Mucograft placement

**Figure 10:**
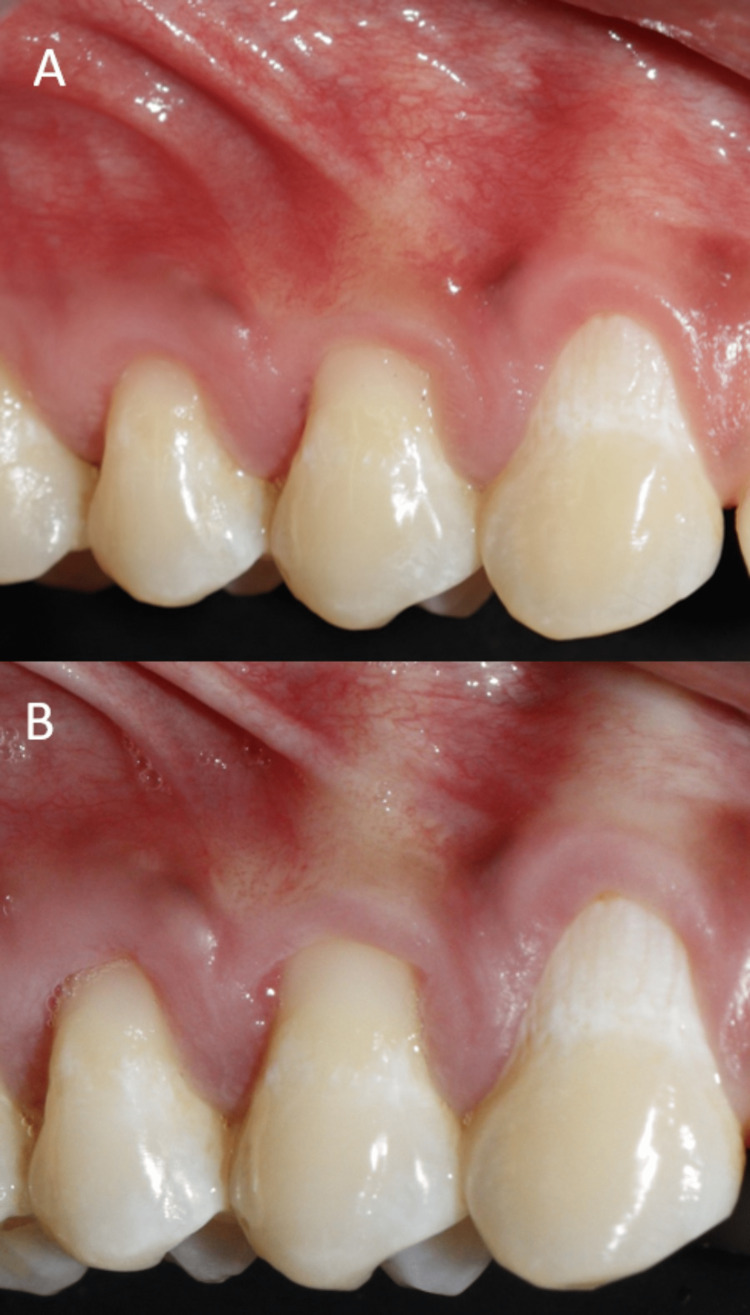
Post-operative images of 14: (A) Six-month post- operative image, (B) one-year post- operative image

Following root conditioning using 24% EDTA (PrefGel) and Emdogain application (Figures 8A, 8B), the graft (Mucograft) was secured on either end with sutures and gradually manipulated into the pouch and through the tunnel, to cover the recipient site. Once the graft was completely inside the tunnel, its ends were secured with 5-0 polypropylene sutures (Figures 9A-9C). Tissue adhesive (Periacryl) have been used to provide optimal soft tissue healing and minimize complications like pain, inflammation and bleeding during the initial phase of healing. The patient was followed up for one year, and RES of 10 out of 10 was obtained at the end of Six Months (Figure 10). However, at the end of one year, a score of seven was obtained suggestive of partial coverage owing to the slight apical migration of the gingival margin.

After both surgeries, the patient was prescribed 500 mg amoxicillin, three times daily for five days, as a systemic antibiotic, a combination of Aceclofenac 100 mg and Paracetamol 325 mg as a pain reliever, and 0.2% Chlorhexidine mouth rinse twice daily, for 14 days as a plaque control measure. Partial root coverage was attained on the maxillary left first premolar and complete root coverage was attained on the maxillary right first premolar, with significant improvement in the gingival biotype in both cases. In both cases, a scalloped contour of the gingiva was observed, with adequate color matching to the adjacent tissues.

## Discussion

GR and its complications such as hypersensitivity, root caries, and tooth loss have led clinical researchers to develop several modifications of surgical techniques that aim at achieving complete root coverage. Evidence has shown that tunneling techniques are as efficacious as SCTG and CAF for recession coverage [11]. Salem et al. reported that the tunneling technique is superior to the traditional CAF procedure in terms of long-term GR coverage [12]. Tunneling techniques have been shown to result in predictable positive outcomes as demonstrated by the results of our study [13]. Our findings are also in accordance with the observations of a previous systematic review [14]. Clinical studies have also investigated tunneling technique outcomes in combination with various biomaterials. In the present case, EMD was used in conjunction with mucograft as an agent for root biomodification as described by Sculean et al. [5]. EMD enhances the beneficial effects of recession coverage by improving soft tissue healing [8]. The significant recession reduction in our case can also be partly attributed to the effect of Emdogain.

Furthermore, in our case, a porcine collagen matrix (mucograft) was used for recession coverage of the right maxillary first premolar thus avoiding the need for a second surgery for graft harvesting thereby reducing patient morbidity. The double-layered structure of mucograft promotes cell migration and increases keratinized tissue thickness, which could be appreciated in our case. Aroca et al., in their study [15], concluded that mucograft could be used as an alternative to SCTG for recession coverage. Our results are in line with those reported by Schmitt et al. in 2016 where they stated that mucograft aids in the augmentation of keratinized tissue [16]. Vincent-Bugnas et al. reported that xenogeneic acellular dermal matrix (Mucoderm®) yielded results comparable to SCTG which also was in alignment with the results of our study [17].

Tissue adhesive (Periacryl) had been applied at the surgical site after suturing in the maxillary right premolar region. It has been proven to provide optimal soft tissue healing and minimize complications like pain, inflammation, and bleeding during the initial phase of healing. It also acts as a protective barrier against food debris. Furthermore, researchers have reported that postoperative pain intensity is much lesser with the application of tissue adhesive [18].

RES is a system used to assess the aesthetic outcome six months after the root coverage procedure [9]. The RES score was calculated for both teeth as we could attain a one-year follow-up, a period adequate to provide soft tissue maturity and stability. In both sites, the length of the recession was reduced from 3 mm to 1 mm along with the change in the periodontal phenotype. The results of our case are comparable with the present literature evidence as suggested by Chambrone et al. in their Cochrane systematic review [19].

In our study, the two techniques utilized for recession coverage were the laterally closed tunnel technique (LCTT) and the pouch and tunnel technique (POTT). Both techniques were described for the management of isolated recession. Based on the type of recession, complete root coverage rates were found to be between 70% and 75% in the LCTT [5]. In this technique, the margins of the pouch were approximated mesially and distally to cover a greater part of the exposed root surface. In the POTT [20], the graft was manipulated into the pouch, through the tunnel, and once completely inside the tunnel, it was positioned coronal to the cementoenamel junction. The advantage of POTT is the preservation of lateral blood supply to the site. Salem et al. reported that POTT resulted in long-term clinical coverage of GR compared to CAF with improved gingival thickness, keratinized tissue, and esthetic results [12]. In our study, both techniques demonstrated an appreciable amount of recession coverage at a one-year follow-up.

However, one of our limitations is that only partial root coverage was achieved in both sites. This might partly be attributed to the inadequate thickness of the SCTG procured from the palate, the lack of complete closure of the recipient site after graft placement, and patient’s tooth-brushing habit.

## Conclusions

The major complication of GR is compromised aesthetics and root hypersensitivity thus resulting in patient discomfort. Hence, the management of GR is very crucial. However, patient and site selection plays a crucial role in determining the ultimate outcome. In addition to the traditional measures used to manage GR, various minimally invasive techniques have been proven successful. Tunneling appears to be an efficacious minimally invasive technique that can achieve satisfactory root coverage. Thus, GR when detected earlier and treated appropriately will result in a predictable outcome.
